# B cell subsets in adult-onset Still’s disease: potential candidates for disease pathogenesis and immunophenotyping

**DOI:** 10.1186/s13075-023-03070-2

**Published:** 2023-06-15

**Authors:** Xiangyu Fang, Hua Ye, Yang Xie, Chaonan Wei, Shuyan Liu, Haihong Yao, Zhanguo Li, Yuan Jia, Fanlei Hu

**Affiliations:** 1 Department of Rheumatology and Immunology, Peking University People’s Hospital, 11 Xizhimen South Street, Beijing, 100044 China; 2 Beijing Key Laboratory for Rheumatism Mechanism and Immune Diagnosis (BZ0135), Beijing, China; 3 State Key Laboratory of Natural and Biomimetic Drugs, School of Pharmaceutical Sciences, Peking University, Beijing, China; 4 Department of Integration of Chinese and Western Medicine, School of Basic Medical Sciences, Peking University, Beijing, China

**Keywords:** Adult-onset Still’s disease, B cell subsets, Clinical correlation, Immunophenotyping

## Abstract

**Background:**

Adult-onset Still’s disease (AOSD) is a systemic autoinflammatory disorder of unknown etiology. B cells are critical participants in different rheumatic diseases, and their roles in AOSD are rarely investigated. This study aimed to unveil the B cell subset features in AOSD and provide evidence for B cell-based diagnosis and targeted therapies of AOSD.

**Methods:**

B cell subsets in the peripheral blood of AOSD patients and healthy controls (HCs) were detected by flow cytometry. Firstly, the frequencies of B cell subsets were compared. Then, the correlation analysis was performed to explore the correlation between B cell subsets and clinical manifestations in AOSD. Finally, unbiased hierarchical clustering was performed to divide AOSD patients into three groups with different B cell subset features, and the clinical characteristics of the three groups were compared.

**Results:**

The frequencies of B cell subsets were altered in AOSD patients. Disease-promoting subsets (such as naïve B cells, double negative B cells (DN B cells), and plasmablasts) increased, and potential regulatory subsets (such as unswitched memory B cells (UM B cells) and CD24^hi^CD27^+^ B cells (B10 cells)) decreased in the peripheral blood of AOSD patients. In addition, the altered B cell subsets in AOSD correlated with the clinical and immunological features, such as immune cells, coagulation features, and liver enzymes. Intriguingly, AOSD patients could be divided into three groups with distinct B cell immunophenotyping: group 1 (naïve B cells-dominant), group 2 (CD27^+^ memory B cells-dominant), and group 3 (precursors of autoantibody-producing plasma cells-dominant). Moreover, these three group patients demonstrated differential manifestations, including immune cells, liver or myocardial enzymes, coagulation features, and systemic score.

**Conclusions:**

B cell subsets are significantly altered in AOSD patients, potentially contributing to the disease pathogenesis. These findings would inspire B cell-based diagnosis and targeted therapies for this refractory disease.

**Supplementary Information:**

The online version contains supplementary material available at 10.1186/s13075-023-03070-2.

## Background

Adult-onset Still’s disease (AOSD) is an uncommon rheumatic disorder of unknown etiology. It is characterized by spiking fever, arthritis, evanescent rash, lymphadenopathy, hepato-splenomegaly, sore throat, neutrophilic leukocytosis, and abnormality of liver enzymes [1]. Additionally, infectious, neoplastic, and other rheumatological disorders should be excluded before diagnosing AOSD [1]. AOSD patients may experience life-threatening complications, including macrophage activation syndrome (MAS), disseminated intravascular coagulopathy (DIC), and thrombotic thrombocytopenic purpura (TTP) [2]. However, the pathogenesis of AOSD is unclear, and more efficient diagnosis and treatment are needed.

After decades of research, both innate and adaptive immune systems have been found to be involved in the occurrence and development of AOSD [3]. However, while the roles of innate immune cells and T cells in AOSD are partially revealed, the features of B cells in AOSD are still barely researched.

B cells are critical immune cells participating in immune responses by producing antibodies, secreting cytokines, and functioning as antigen-presenting cells [4]. Several significant B cell subsets are defined through their unique functions in immune responses [5, 6]. The crucial roles of these B cell subsets have been revealed in different rheumatic diseases such as rheumatoid arthritis [7], systemic lupus erythematosus [8], and Sjögren’s syndrome [9]. B cell subsets also involve in ankylosing spondylitis and other autoinflammatory syndromes [10, 11]. A recent study classified AOSD patients into three groups through the peripheral immune cell profiles, showing a significant difference in total B cell frequencies among groups [12]. In addition, refractory AOSD patients with or without complications were reported to be successfully treated by a B cell depleting agent (rituximab) [13–17]. However, B cell subset characteristics in AOSD and the mechanisms behind rituximab treating AOSD need to be further studied.

In this study, we compared B cell features between AOSD patients and HCs and preliminarily defined the relationship between B cell subsets and clinical manifestations of AOSD patients. We also classified AOSD patients into three groups by hierarchical cluster of B cell subsets. The results may inspire further exploration of the roles of B cells in the pathogenesis, diagnosis, and targeted therapies of AOSD.

## Methods

### Patients and controls

From April 2019 to March 2021, 27 AOSD patients who met Yamaguchi criteria [18] were enrolled in this study from the Department of Rheumatology and Immunology, Peking University People’s Hospital, China. In addition, 40 healthy controls (HCs) were recruited from the physical examination center. Age (31.0 (16 ~ 66) vs 31.5 (26 ~ 65), *P* = 0.424, Mann–Whitney *U* test) and sex (26/1 vs 35/5, *χ*^2^ (continuity correction) = 0.641, *P* = 0.423) of HCs and AOSD patients were matched. Our study was approved by the Institutional Medical Ethics Review Board of Peking University People’s Hospital. All participants gave informed consent to donate their blood samples and clinical data for research, and their personal information remained confidential.

### Clinical data and systemic feature score

Clinical data were obtained from medical records systems. Patients’ information was recorded, including age, sex, disease duration, and clinical manifestations (symptoms, signs, and laboratory variables). Indexes and ratios (SII (systemic immune-inflammation index, PLT × neutrophil count/lymphocyte count), CAR (CRP/ALB ratio), PNI (prognostic nutritional index, albumin + 0.005 × peripheral lymphocyte count), FER (ferritin/ESR ratio), NLR (neutrophil count/lymphocyte count ratio), and LAR (LDH/ALB ratio)) studied in previous research about AOSD were also calculated [19–22]. A modified Pouchot systemic score (mPss) comprising 12 disease manifestations (fever, evanescent rashes, sore throat, arthritis, myalgia, pleuritis, pericarditis, pneumonitis, lymphadenopathy, hepatomegaly or abnormal liver function tests, elevated leukocyte count > 15,000/μL, and serum ferritin > 3000 μg/L) was used to assess the disease activity and patients whose mPss ≥ 4 were defined as active patients [23]. The statistic of the above data is shown in Table 1.

**Table 1:** Patient characteristics

Characteristics	Active (*n* = 18)	Inactive (*n* = 9)
**Basic information**		
Sex, no. female/no. male	17/1	9/0
Age, median (range), years	35 (18 − 66)	31 (18 − 62)
Duration, median (range), years	0.71 (0.04 − 16)	0.67 (0.25 − 1.1)
**Clinical features**		
Fever (*n*, %)	10/18 (55.6)	0/9 (0.0)
Arthritis (*n*, %)	7/18 (38.9)	0/9 (0.0)
Skin rash (*n*, %)	12/18 (66.7)	2/9 (22.2)
Sore throat (*n*, %)	6/18 (33.3)	0/9 (0.0)
Splenomegaly (*n*, %)	6/18 (33.3)	3/9 (33.3)
Lymphadenopathy (*n*, %)	11/18 (61.1)	2/9 (22.2)
Hepatomegaly (*n*, %)	1/18 (5.6)	3/9 (33.3)
Serositis (*n*, %)	7/18 (38.9)	2/9 (22.2)
mPss, median (range)	5.5 (4 − 9)	1 (1 − 3)
MAS (*n*, %)	5/18 (27.8)	0/9 (0.0)
**Laboratory features**		
WBC, × 10^9^/L	13.1 (10.4, 14.3)	7.4 (6.2, 8.3)
Percentage of neutrophils, %	81.3 (73.5, 86.4)	64.9 (56.3, 71.3)
Percentage of lymphocytes, %	12.4 (9.5, 15.4)	25.4 (22.6, 33.8)
Percentage of monocytes, %	4.3 (2.9, 6.5)	7.8 (5.7, 8.9)
Neutrophil count, × 10^9^/L	9.8 (8.3, 12.5)	5.2 (3.6, 5.4)
Lymphocyte count, × 10^9^/L	1.7 (0.9, 2.0)	2.0 (1.9, 2.1)
Monocyte count, × 10^9^/L	0.5 (0.4, 0.8)	0.6(0.4, 0.7)
Percentage of T cells, %	77.65 (70.7, 79.9)	76.8 (70.5, 81.6)
Percentage of B cells, %	5.3 (3.7, 7.7)	9.4 (4.3, 12.2)
Percentage of NK cells, %	5.5 (2.7, 8.0)	4.3 (3.2, 9.8)
Hb, g/L	108.0 (93.8, 123.3)	120.0 (112.0, 124.0)
PLT, × 10^9^/L	308.5 (261.3, 347.8)	300.0 (263.0, 357.0)
ALT, U/L	42.0 (14.3, 88)	16.0 (10.0, 21.0)
AST, U/L	36.0 (21.3, 73.5)	15.0 (15.0, 17.0)
AST/ALT	1.4 (0.6, 1.9)	1.0 (0.7, 1.5)
GGT, U/L	47.0 (34.0, 107.5)	16.0 (12.0, 20.5)
LDH, U/L	387.0 (297.8, 507.0)	206.0 (177.0, 226.0)
ALP, U/L	95.0 (87.5, 144.0)	62.0 (51.0, 73.0)
HBDH, U/L	232.0 (190.5, 298.3)	151.0 (122.0, 163.0)
TBIL, mmol/L	8.0 (6.7, 12.1)	12.2 (7.8, 14.9)
CBIL, mmol/L	2.9 (2.5, 4.4)	3.6 (2.5, 4.8)
TG, mmol/L	1.4 (1.1, 2.1)	1.2 (1.0, 1.9)
TP, g/L	64.2 (61.1, 70.2)	67.6 (63.2, 68.6)
ALB, g/L	33.5 (29.9, 35.5)	39.7 (35.0, 41.3)
GLB, g/L	32.4 (27.5, 34.8)	27.8 (26.0, 28.7)
AGR	1.0 (0.9, 1.3)	1.3 (1.2, 1.5)
Fibrinogen, g/L	416.5 (290.5, 502.3)	308.5 (244.3, 350.0)
D-Dimer, μg/L	478.0 (289.0, 651.0)	102.5 (47.5, 168.3)
PT, sec	12.6 (11.9, 12.8)	11.7 (11.2, 12.3)
APTT, sec	29.1 (26.0, 30.3)	29.5 (28.0, 31.1)
ESR, mm/H	46.5 (18.5, 69.5)	13.0 (8.0, 29.0)
CRP, mg/L	47.1 (21.2, 89.5)	3.7 (0.5, 5.6)
Ferritin, μg/L	2611.5 (960.8, 5003.3)	37.5 (16.9, 354.7)
RF (*n*, %)	1/18 (5.6)	0/9 (0.0)
ANA ≥ 1: 80 (*n*, %)	2/18 (11.1)	0/9 (0.0)
ACPA (*n*, %)	0/18 (0.0)	0/18 (0.0)
**Index and ratios**		
SII	1991.6 (1146.6, 3373.7)	1028.5 (560.3, 1937.5)
CAR	1.4 (0.6, 2.5)	0.2 (0.0, 0.7)
PNI	41.6 (37.5, 44.3)	45.6 (42.5, 50.2)
FER	48.6 (28.7, 96.8)	25.4 (8.4, 56.8)
NLR	6.7 (4.0, 10.1)	3.4 (2.5, 6.3)
LAR	11.3 (8.2, 14.9)	8.0 (5.4, 9.6)
**Medications**		
NSAIDs (*n*, %)	8/18 (44.4)	7/9 (77.8)
Corticosteroids (*n*, %)	17/18 (94.4)	9/9 (100.0)
Traditional DMARDs (*n*, %)	11/18(61.1)	8/9 (88.9)
Biological DMARDs (*n*, %)	5/18 (27.8)	1/9 (11.1)

Unless otherwise specified, values are the medians (interquartile ranges). Values were rounded to one decimal place

*mPss* Modified Pouchot score, *MAS* Macrophage activation syndrome, *WBC* White blood cell, *Hb* Hemoglobin, *PLT* Platelet count, *ALT* Alanine aminotransferase, *AST* Aspartate aminotransferase, *GGT* γ-glutamyl transpeptidase, *LDH* Lactate dehydrogenase, *ALP* Alkaline phosphatase, *HBDH* Hydroxybutyrate dehydrogenase, *TBIL* Total bilirubin, *CBIL* Conjugated bilirubin, *TG* Triglycerides, *TP* Total protein, *ALB* Albumin, *GLB* Globulin, *AGR* Albumin/globulin ratio, *PT* Prothrombin time, *APTT* Activated partial thromboplastin time, *ESR* Erythrocyte sedimentation rate, *CRP* C-reactive protein, *RF* Rheumatoid factor, *ANA* Antinuclear antibody, *ACPA* Anti-cyclic citrullinated peptide antibody, *SII* Systemic immune-inflammation index (PLT × neutrophil count/lymphocyte count), *CAR* CRP/ALB ratio, *PNI* Prognostic nutritional index (albumin + 0.005 × peripheral lymphocyte count), *FER* Ferritin/ESR ratio, *NLR* Neutrophil count/lymphocyte count ratio, *LAR* LDH/ALB ratio

### Sample processing and flow cytometry

Fresh collected whole blood was obtained and processed within 24 h. Fluorescein-labeled antibodies (anti-CD3-PerCP-Cy5.5, anti-CD19-APC-Cy7, anti-CD20-PE-Cy7, anti-CD27-APC, anti-CD24-FITC, anti-IgD-Pacific Blue) were incubated in 200 μL whole blood for 30 min in the dark. Then, 3 mL Lysing Solution was added for 10 min to lyse the erythrocytes. Then, samples were centrifuged at 1800 rpm for 5 min, and the supernatants were removed. Finally, the stained samples were washed in phosphate-buffered saline and stored at 4 °C before flow detection.

**Fig. 1:**
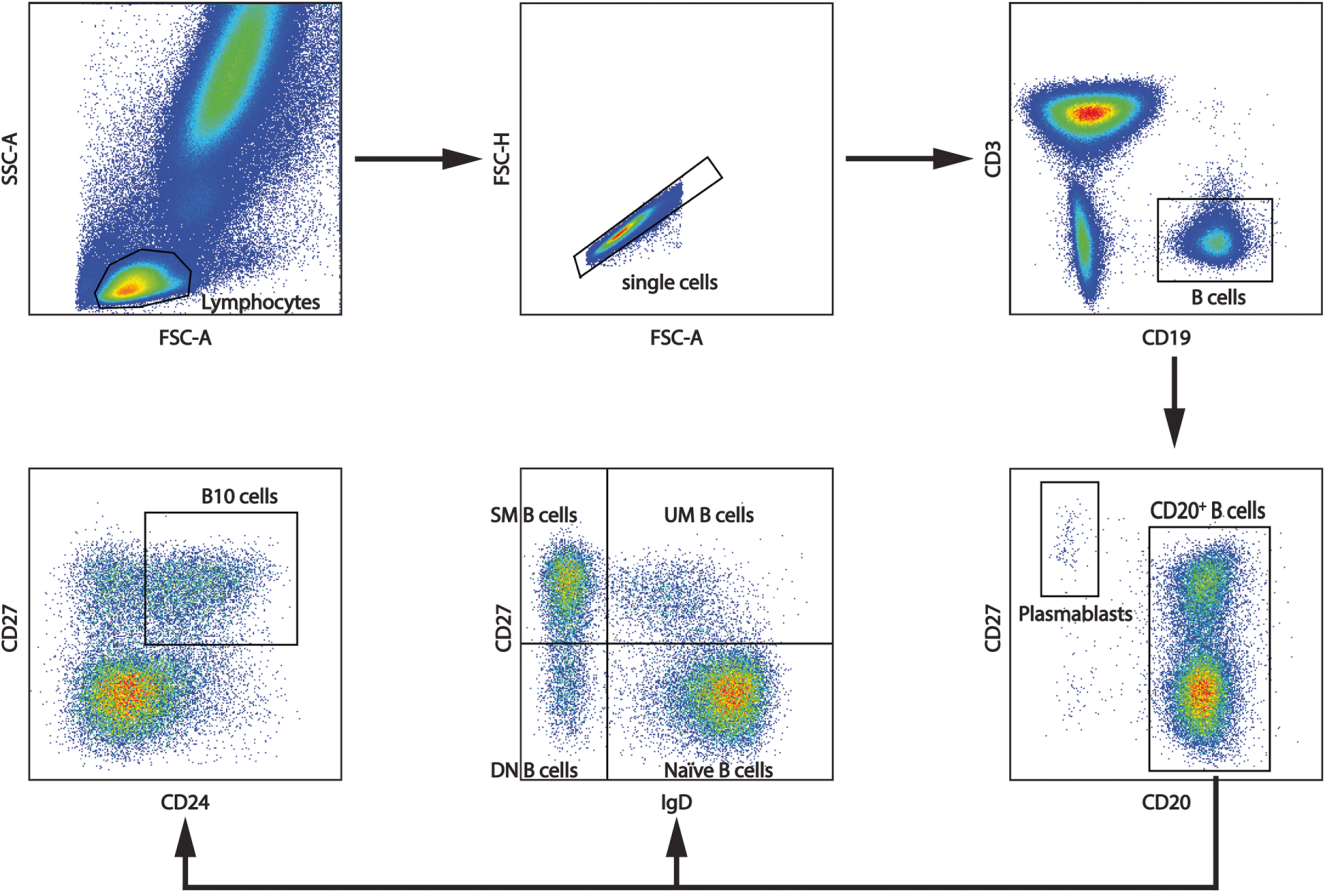
Gating strategy for flow cytometry analyses of the frequencies of B cell subsets. After lymphocyte gating according to FSC and SSC, CD3^−^CD19^+^ lymphocytes were selected and resolved into subsets by analyzing CD20, CD24, CD27, or IgD expression

B cell subsets, including IgD^+^CD27^−^ naïve B cells (naïve B cells), IgD^+^CD27^+^ unswitched memory B cells (UM B cells), IgD^−^CD27^+^ switched memory B cells (SM B cells), total CD27^+^ memory B cells, IgD^−^CD27^−^ double negative B cells (DN B cells), CD19^+^CD20^−^CD27^hi^ plasmablasts (plasmablasts), and CD24^hi^CD27^+^ B cells (B10 cells) [5, 24] were detected on the FACSAria II flow cytometer. The results were analyzed with FlowJo v10 (TreeStar, Woodburn, OR). The strategies for gating are shown in Fig. 1.

### Principle component analysis and unbiased cluster analysis

Principal component analysis (PCA) and unbiased cluster analysis were performed following the method in the research of Ruru Guo et al. [12]. PCA was conducted to explore the difference in peripheral B cell subsets features between patients with AOSD and HCs. Then, unbiased hierarchical clustering for AOSD patients based on B cell subsets (naïve B cell%, UM B cell%, SM B cell%, DN B cell%, plasmablast%, B10 cell%) was analyzed using Ward’s method.

### Statistical analysis

Flow cytometry data of HCs and AOSD patients were concatenated and analyzed using FlowJo v10 plugins, including down-samples and t-SNE. Statistical calculations were performed using SPSS 25.0 (SPSS Inc., Chicago, IL). Data that adhered to the normal distribution and had a common variance were evaluated by Student’s *t* test or one-way ANOVA; data that did not adhere to the normal distribution or did not have a common variance were evaluated by Mann–Whitney *U* test or Kruskal–Wallis *H* test. Uncorrected Fisher’s LSD test or uncorrected Dunn’s test were used for post hoc multiple comparisons. The correlation between indicators was evaluated by Spearman’s rank correlation test. *P* values < 0.05 were considered statistically significant: *P < 0.05, **P < 0.01, ***P < 0.001, and ns, no significance.

## Results

### Altered frequencies of B cell subsets in AOSD

**Fig. 2:**
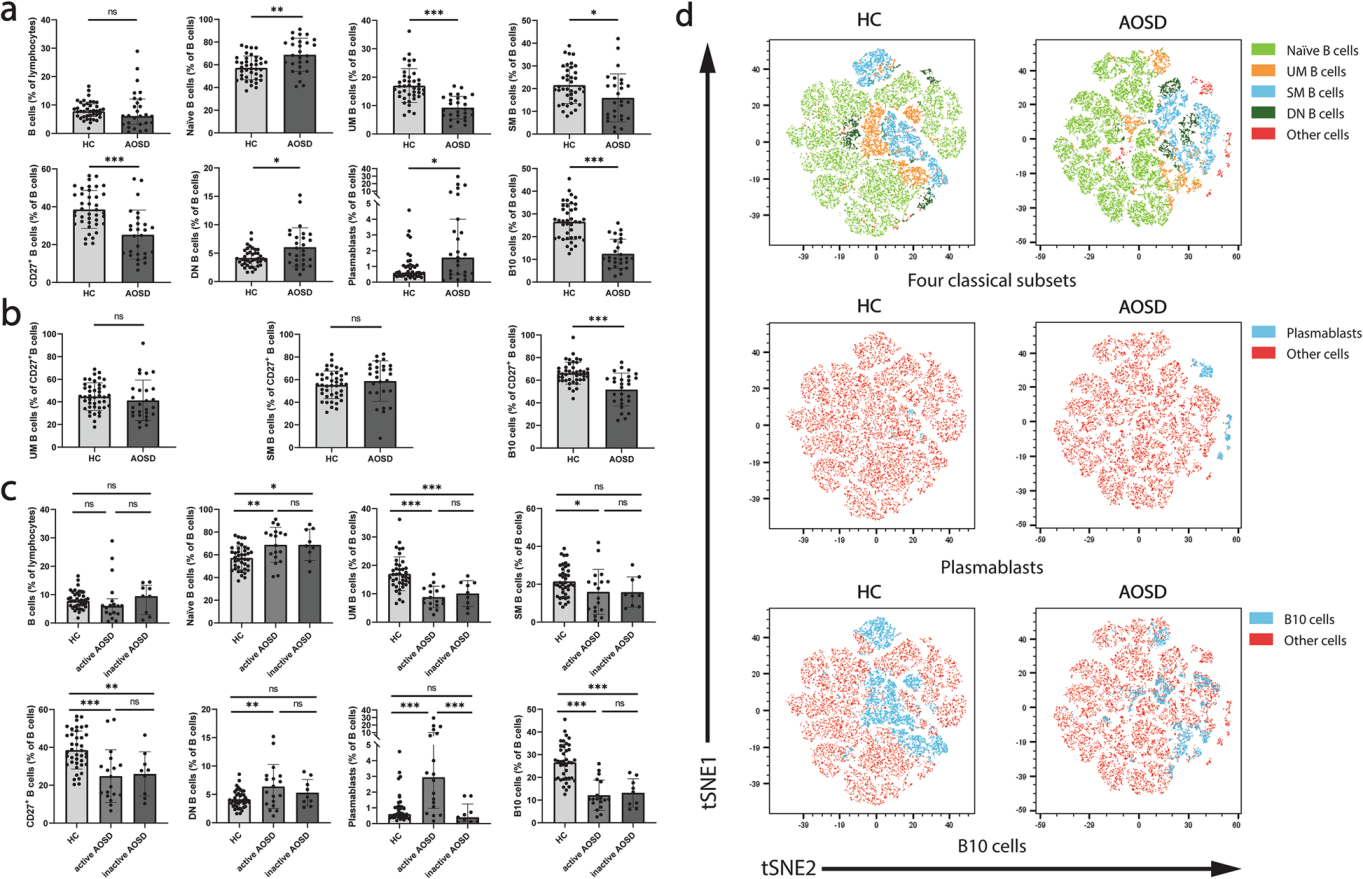
Altered frequencies of B cell subsets in AOSD PBMC. **a** The frequencies of B cell subsets in the peripheral blood of the healthy controls (*n* = 40) and AOSD patients (*n* = 27). **b** The relative frequencies of three CD27^+^ B cell subsets in the total CD27^+^ B cells. **c** The frequencies of B cell subsets in the peripheral blood of the healthy controls (*n* = 40), active AOSD patients (*n* = 18), and inactive AOSD patients (*n* = 9). **d** t-SNE analyses of B cell subsets in healthy controls’ and AOSD patients’ peripheral blood. *P < 0.05, **P < 0.01, ***P < 0.001, and ns, no significance (unpaired *t* test, naïve B cells, UM B cells, SM B cells, CD27^+^ B cells, DN B cells, and B10 cells in **a **and** b**; Mann–Whitney *U* test, B cells, and plasmablasts in **a**; One-way ANOVA followed by uncorrected Fisher’s LSD test, naïve B cells, UM B cells, SM B cells, CD27^+^ B cells, DN B cells and B10 cells in **c**; Kruskal–Wallis *H* test followed by uncorrected Dunn’s test, B cells and plasmablasts in **c**)

We compared the frequencies of B cell subsets (including IgD^+^CD27^−^ naïve B cells (naïve B cells), IgD^+^CD27^+^ unswitched memory B cells (UM B cells), IgD^−^CD27^+^ switched memory B cells (SM B cells), total CD27^+^ memory B cells, IgD^−^CD27^−^ double negative B cells (DN B cells), CD19^+^CD20^−^CD27^hi^ plasmablasts (plasmablasts), and CD24^hi^CD27^+^ B cells (B10 cells)) between AOSD patients and HCs. We found that the frequencies of B cell subsets altered in AOSD patients (Fig. 2a). These alterations included a decrease in UM B cells (9.28 vs 17.03, *P* < 0.0001), SM B cells (15.92 vs 21.54, *P* = 0.0162), total CD27^+^ memory B cells (25.21 vs 38.57, *P* < 0.0001), and B10 cells (12.50 vs 26.61, *P* < 0.0001) and an increase in naïve B cells (68.79 vs 57.27, *P* = 0.0004), DN B cells (6.04 vs 4.16, *P* = 0.0034), and plasmablasts (1.56 vs 0.61, *P* = 0.0412). Although there was no significant change in total B cells, the dispersion degree of total B cells in AOSD patients was higher than in HCs (interquartile range: 8.87 vs 4.46).

We also compared the relative frequencies of three CD27^+^ B cell subsets in the total CD27^+^ B cells. As shown in Fig. 2b, the frequency of B10 cells was significantly lower in AOSD patients than HCs. There was no significant change in UM B cells and SM B cells in AOSD patients compared to HCs.

Then, AOSD patients were divided into two groups (the active group and the inactive group) according to mPss, and the frequencies of B cell subsets were compared again (Fig. 2c). A significantly higher plasmablasts was found in the active group compared to the inactive group (2.94 vs 0.40, *P* = 0.0018). In addition, the active group had significantly higher DN B cells and significantly lower SM B cells than HCs, while the inactive group did not. These results indicated that the alterations of DN B cells and plasmablasts were more significant in the active group than in the inactive group.

Moreover, t-SNE analys1s was also used to more intuitively demonstrate the altered B cell subsets’ frequencies (Fig. 2d). A different B cell subsets distribution also was found between AOSD patients and HCs after being compared by PCA (Fig. S1a, b). In addition, the active AOSD patients had a more significant change in B cell subsets phenotype than the inactive ones.

### Correlation of B cell subsets with clinical manifestations of AOSD patients

**Fig. 3:**
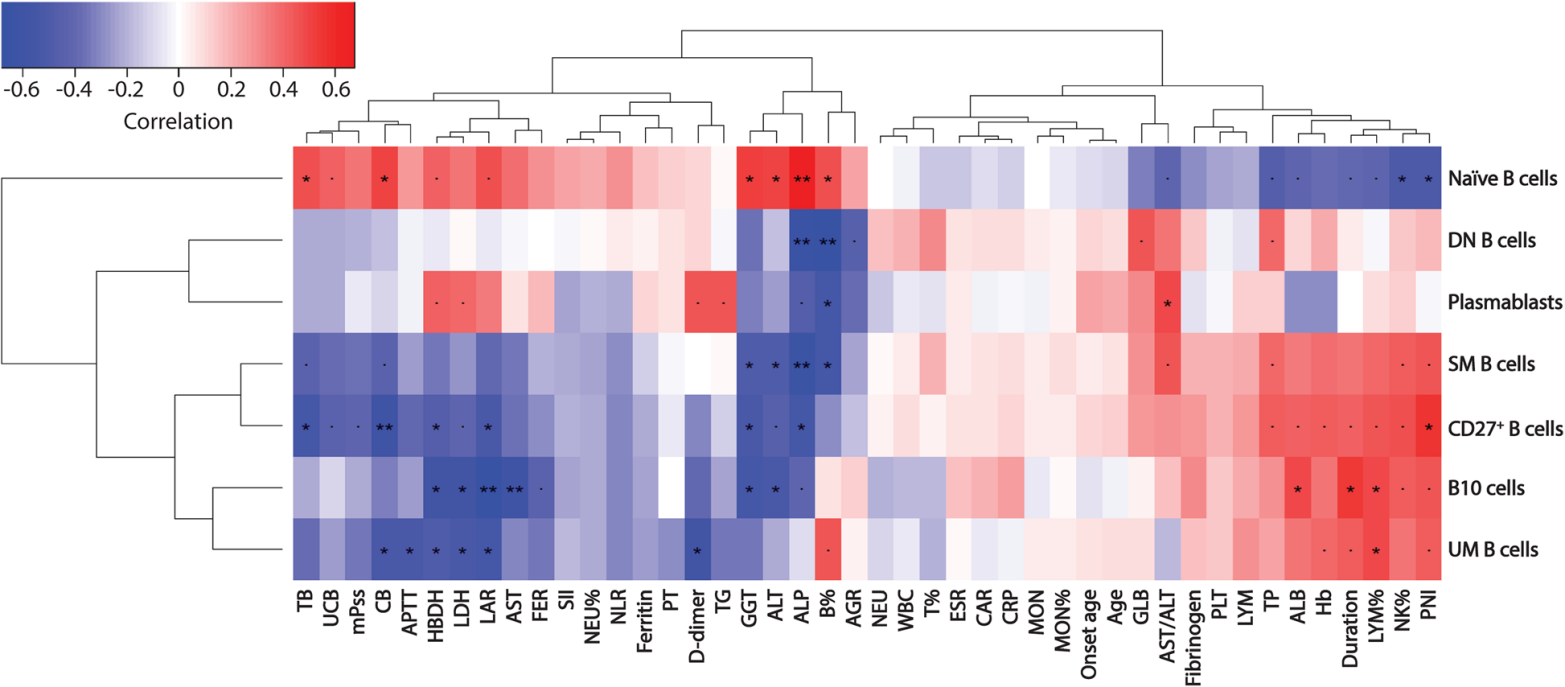
Correlation analysis of B cell subsets with clinical manifestations of AOSD patients. Correlation matrix among B cell subsets and clinical manifestations. *P < 0.05, **P < 0.01, and ‘.’ marginal significance (Spearman’s rank correlation test)

Since the active group had a more significant alteration in B cell subset features than the inactive group, it was chosen in the correlation analysis to ensure the typicality of AOSD samples. The potential correlations between the frequencies of B cell subsets and clinical manifestations were analyzed (Table 2 and Fig. 3).

**Table 2:** Correlation between B cell subsets and clinical manifestations

Clinical manifestations	Naïve B cells		UM B cells		SM B cells		CD27^+^ B cells		DN B cells		Plasmablasts		B10 cells	
	*r*	*p*	*r*	*p*	*r*	*p*	*r*	*p*	*r*	*p*	*r*	*p*	*r*	*p*
Duration	− 0.406	0.095	0.432	0.074	0.361	0.142	0.419	0.083	0.057	0.823	0.007	0.977	0.553^*^	**0.017**
Age	− 0.109	0.668	0.097	0.701	0.065	0.799	0.119	0.639	0.091	0.720	0.216	0.389	− 0.037	0.883
Age of onset	− 0.092	0.717	0.077	0.760	0.047	0.852	0.110	0.663	0.102	0.687	0.243	0.332	− 0.075	0.766
mPss	0.363	0.139	− 0.351	0.154	− 0.367	0.135	− 0.419	0.084	− 0.196	0.435	− 0.048	0.851	− 0.214	0.393
WBC	− 0.044	0.861	− 0.055	0.829	0.062	0.807	0.088	0.729	0.191	0.448	− 0.061	0.810	− 0.187	0.457
NEU	0.001	0.997	− 0.106	0.675	0.018	0.945	0.030	0.906	0.160	0.526	− 0.150	0.553	− 0.198	0.432
NEU%	0.224	0.372	− 0.220	0.381	− 0.228	0.362	− 0.214	0.395	0.013	0.958	− 0.193	0.443	− 0.216	0.389
LYM	− 0.224	0.371	0.297	0.232	0.213	0.396	0.279	0.262	− 0.066	0.794	0.126	0.618	0.220	0.381
LYM%	− 0.422	0.081	0.488^*^	**0.040**	0.391	0.108	0.451	0.060	− 0.022	0.932	0.094	0.711	0.485^*^	**0.041**
MON	− 0.001	0.997	0.061	0.810	0.049	0.848	0.045	0.858	0.049	0.848	− 0.035	0.890	− 0.062	0.807
MON%	− 0.049	0.848	0.050	0.845	0.090	0.723	0.045	0.858	0.032	0.900	0.042	0.868	0.024	0.925
T cells%	− 0.146	0.565	− 0.197	0.433	0.202	0.421	0.032	0.900	0.315	0.203	− 0.075	0.766	− 0.183	0.467
B cells%	0.473^*^	**0.047**	0.449	0.062	− 0.543^*^	**0.020**	− 0.286	0.250	− 0.669^**^	**0.002**	− 0.540^*^	**0.021**	0.072	0.775
NK cells%	− 0.513^*^	**0.030**	0.366	0.135	0.437	0.070	0.465	0.052	0.146	0.565	0.131	0.604	0.427	0.077
Hb	− 0.382	0.118	0.400	0.100	0.383	0.116	0.428	0.076	0.216	0.389	− 0.285	0.251	0.354	0.149
PLT	− 0.235	0.347	0.152	0.548	0.207	0.411	0.208	0.409	− 0.033	0.896	− 0.019	0.942	0.138	0.586
ALT	0.501^*^	**0.034**	− 0.220	0.381	− 0.483^*^	**0.042**	− 0.451	0.060	− 0.164	0.515	− 0.261	0.295	− 0.528^*^	**0.024**
AST	0.386	0.114	− 0.310	0.210	− 0.342	0.165	− 0.370	0.131	− 0.013	0.958	0.076	0.763	− 0.591^**^	**0.010**
AST/ALT	− 0.414	0.088	− 0.179	0.478	0.455	0.058	0.292	0.240	0.354	0.150	0.488^*^	**0.040**	0.154	0.542
GGT	0.533^*^	**0.023**	− 0.339	0.169	− 0.495^*^	**0.037**	− 0.530^*^	**0.024**	− 0.357	0.146	− 0.320	0.195	− 0.537^*^	**0.022**
LDH	0.360	0.142	− 0.534^*^	**0.023**	− 0.277	0.266	− 0.414	0.088	0.009	0.971	0.408	0.093	− 0.578^*^	**0.012**
ALP	0.669^**^	**0.002**	− 0.085	0.738	− 0.627^**^	**0.005**	− 0.547^*^	**0.019**	− 0.652^**^	**0.003**	− 0.466	0.051	− 0.418	0.084
HBDH	0.411	0.090	− 0.493^*^	**0.038**	− 0.346	0.159	− 0.471^*^	**0.048**	− 0.059	0.816	0.411	0.090	− 0.573^*^	**0.013**
TG	0.023	0.929	− 0.344	0.162	0.018	0.945	− 0.135	0.593	− 0.024	0.925	0.458	0.056	− 0.244	0.329
TBIL	0.480^*^	**0.044**	− 0.375	0.126	− 0.427	0.077	− 0.540^*^	**0.021**	− 0.224	0.372	− 0.220	0.381	− 0.210	0.403
CBIL	0.499^*^	**0.035**	− 0.526^*^	**0.025**	− 0.426	0.078	− 0.595^**^	**0.009**	− 0.167	0.507	− 0.111	0.662	− 0.329	0.182
UCBIL	0.416	0.086	− 0.248	0.321	− 0.382	0.118	− 0.441	0.067	− 0.224	0.371	− 0.223	0.373	− 0.105	0.680
TP	− 0.468	0.050	0.227	0.365	0.405	0.096	0.436	0.071	0.407	0.094	0.126	0.618	0.287	0.249
ALB	− 0.412	0.089	0.367	0.134	0.339	0.169	0.423	0.081	0.105	0.677	− 0.287	0.248	0.498^*^	**0.035**
GLB	− 0.326	0.186	0.083	0.745	0.282	0.257	0.277	0.266	0.459	0.055	0.299	0.227	0.039	0.877
AGR	0.228	0.363	0.034	0.893	− 0.233	0.351	− 0.166	0.510	− 0.445	0.064	− 0.383	0.117	0.129	0.609
PT	0.123	0.638	− 0.290	0.259	− 0.026	0.922	− 0.052	0.844	0.073	0.782	0.068	0.797	0.000	1.000
APTT	0.275	0.286	− 0.520^*^	**0.033**	− 0.251	0.330	− 0.400	0.112	− 0.029	0.911	− 0.034	0.896	− 0.260	0.313
D-dimer	0.118	0.653	− 0.576^*^	**0.016**	− 0.009	0.974	− 0.314	0.220	0.110	0.673	0.446	0.073	− 0.403	0.109
Fibrinogen	− 0.284	0.254	0.168	0.505	0.200	0.425	0.267	0.284	0.139	0.581	− 0.075	0.766	0.303	0.222
ESR	− 0.137	0.586	0.021	0.935	0.061	0.810	0.067	0.791	0.081	0.750	0.052	0.839	0.158	0.530
CRP	− 0.121	0.633	− 0.030	0.906	0.098	0.699	0.108	0.669	0.088	0.729	− 0.018	0.945	0.257	0.302
Ferritin	0.170	0.499	− 0.259	0.299	− 0.130	0.607	− 0.214	0.395	0.044	0.861	0.104	0.681	− 0.233	0.353
SII	0.230	0.358	− 0.176	0.484	− 0.214	0.394	− 0.201	0.423	− 0.020	0.938	− 0.245	0.328	− 0.229	0.362
CAR	− 0.086	0.735	− 0.046	0.855	0.071	0.779	0.075	0.766	0.096	0.705	− 0.030	0.906	0.206	0.413
PNI	− 0.503^*^	**0.034**	0.441	0.067	0.461	0.054	0.556^*^	**0.017**	0.185	0.463	− 0.020	0.938	0.454	0.058
FER	0.296	0.233	− 0.344	0.163	− 0.198	0.430	− 0.296	0.233	0.001	0.997	0.174	0.489	− 0.431	0.074
LAR	0.467	0.050	− 0.548^*^	**0.019**	− 0.394	0.105	− 0.529^*^	**0.024**	− 0.049	0.848	0.348	0.157	− 0.679^**^	**0.002**
NLR	0.286	0.250	− 0.304	0.219	− 0.281	0.259	− 0.304	0.219	0.063	0.804	− 0.214	0.395	− 0.311	0.209

The data was analyzed by Spearman’s correlation coefficient test

*Abbreviations*: *mPss* Modified Pouchot score, *WBC* White blood cell, *NEU* Neutrophils, *LYM* Lymphocytes, *MON* Monocytes, *Hb* Hemoglobin, *PLT* Platelet count, *ALT* Alanine aminotransferase, *AST* Aspartate aminotransferase, *GGT* γ-glutamyl transpeptidase, *LDH* Lactate dehydrogenase, *ALP* Alkaline phosphatase, *HBDH* Hydroxybutyrate dehydrogenase, *TBIL* Total bilirubin, *CBIL* Conjugated bilirubin, *UCBIL* Unconjugated bilirubin, *TG* Triglycerides, *TP* Total protein, *ALB* Albumin, *GLB* Globulin, *AGR* Albumin/globulin ratio, *PT* Prothrombin time, *APTT* Activated partial thromboplastin time, *ESR* Erythrocyte sedimentation rate, *CRP* C-reactive protein, *SII* Systemic immune-inflammation index (PLT × neutrophil count/lymphocyte count), *CAR* CRP/ALB ratio, *PNI* Prognostic nutritional index (albumin + 0.005 × peripheral lymphocyte count), *FER* Ferritin/ESR ratio, *LAR* LDH/ALB ratio, *NLR* Neutrophil count/lymphocyte count ratio

^*^P < 0.05

^**^P < 0.01

Firstly, the correlations between B cell subsets and immune cells were analyzed. Naïve B cells positively correlated with B% and negatively correlated with NK% and LYM%. UM B cells positively correlated with LYM% and B%. SM B cells positively correlated with NK% and negatively correlated with B%. Total CD27^+^ memory B cells positively correlated with LYM% and NK%. DN B cell and plasmablast negatively correlated with B%.

Then, the coagulation parameters, including prothrombin time (PT), activated partial thromboplastin time (APTT), fibrinogen, and D-dimer, were studied because coagulation disorder may occur in AOSD. UM B cells negatively correlated with APTT and D-dimer, although all the patients’ APTT values were in the normal range. Conversely, plasmablasts positively correlated with D-dimer.

The correlations between B cell subsets and liver and myocardial enzymes were also studied. Naïve B cells positively correlated with alanine transaminase (ALT), γ-glutamyl transferase (GGT), alkaline phosphatase (ALP), and hydroxybutyrate dehydrogenase (HBDH) and negatively correlated with AST/ALT ratio. UM B cells negatively correlated with lactate dehydrogenase (LDH) and HBDH. SM B cells positively correlated with AST/ALT ratio and negatively correlated with ALT, GGT, and ALP. Total CD27^+^ memory B cells negatively correlated with ALT, GGT, LDH, ALP, and HBDH. DN B cells negatively correlated with ALP. Plasmablast positively correlated with AST/ALT ratio, LDH, and HBDH and negatively correlated with ALP.

Moreover, the correlations between B cell subsets and other liver function indicators were studied. For example, naïve B cells positively correlated with bilirubins, while UM B cells and total CD27^+^ memory B cells negatively correlated with bilirubin. In addition, B10 cells positively correlated with serum albumin (ALB), while B10 cells, UM B cells, and total CD27^+^ memory B cells negatively correlated with LDH/ALB ratio.

In inflammation-related indexes and ratios, naïve B cells negatively correlated with the prognostic nutritional index (PNI), while UM B cells, SM B cells, total CD27^+^ memory B cells, and B10 cells positively correlated with PNI. Surprisingly, B10 cells and UM B cells positively correlated with disease duration.

The 18 active AOSD patients were also divided into two groups with or without a specific clinical manifestation (fever, arthritis, skin rash, sore throat, splenomegaly, lymphadenopathy, and serositis). The frequencies of B cell subsets in the two subgroups were compared (Table S1). The results showed that the frequencies of total CD27^+^ memory B cells decreased in AOSD patients with a sore throat.

### Unbiased cluster analysis of AOSD B cell subsets

**Fig. 4:**
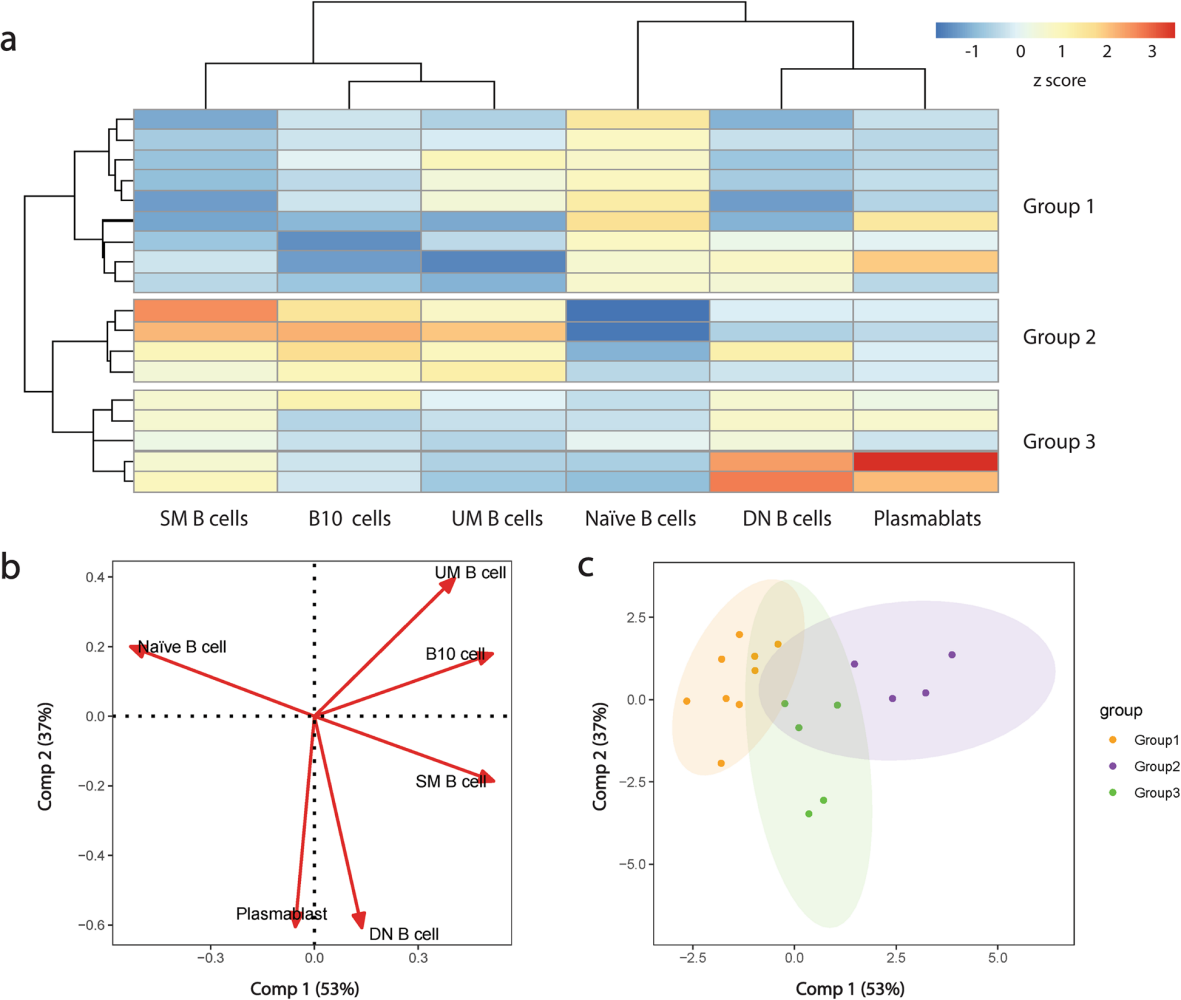
Unbiased cluster analysis of B cell subsets in AOSD. **a** Hierarchical cluster analysis divided patients with active AOSD (*n* = 18) into three subgroups. **b** Results of PCA based on B cell subsets in AOSD patients, first and second principal components were chosen to virtualize different B cell subsets. **c** Comp1 and Comp 2 values in individual patients with AOSD

After unbiased cluster analysis, patients with active AOSD were divided into three groups (group 1 (naïve B cells-dominant group), group 2 (CD27^+^ memory B cells-dominant group), and group 3 (precursors of autoantibody-producing plasma cells-dominant group)) according to B cell subsets features (Fig. 4a). According to the clustering results, six B cell subset variables can also be divided into three groups (naïve B cell subset group, precursors of autoantibody-producing plasma cells (DN B cells and plasmablasts) group, and CD27^+^ memory B cell subsets (UM B cells, SM B cells, and B10 cells) group). In addition, PCA was also conducted, and the PCA score plot confirmed that patients with AOSD were separated into group 1, group 2, and group 3 (Fig. 4b, c).

**Fig. 5:**
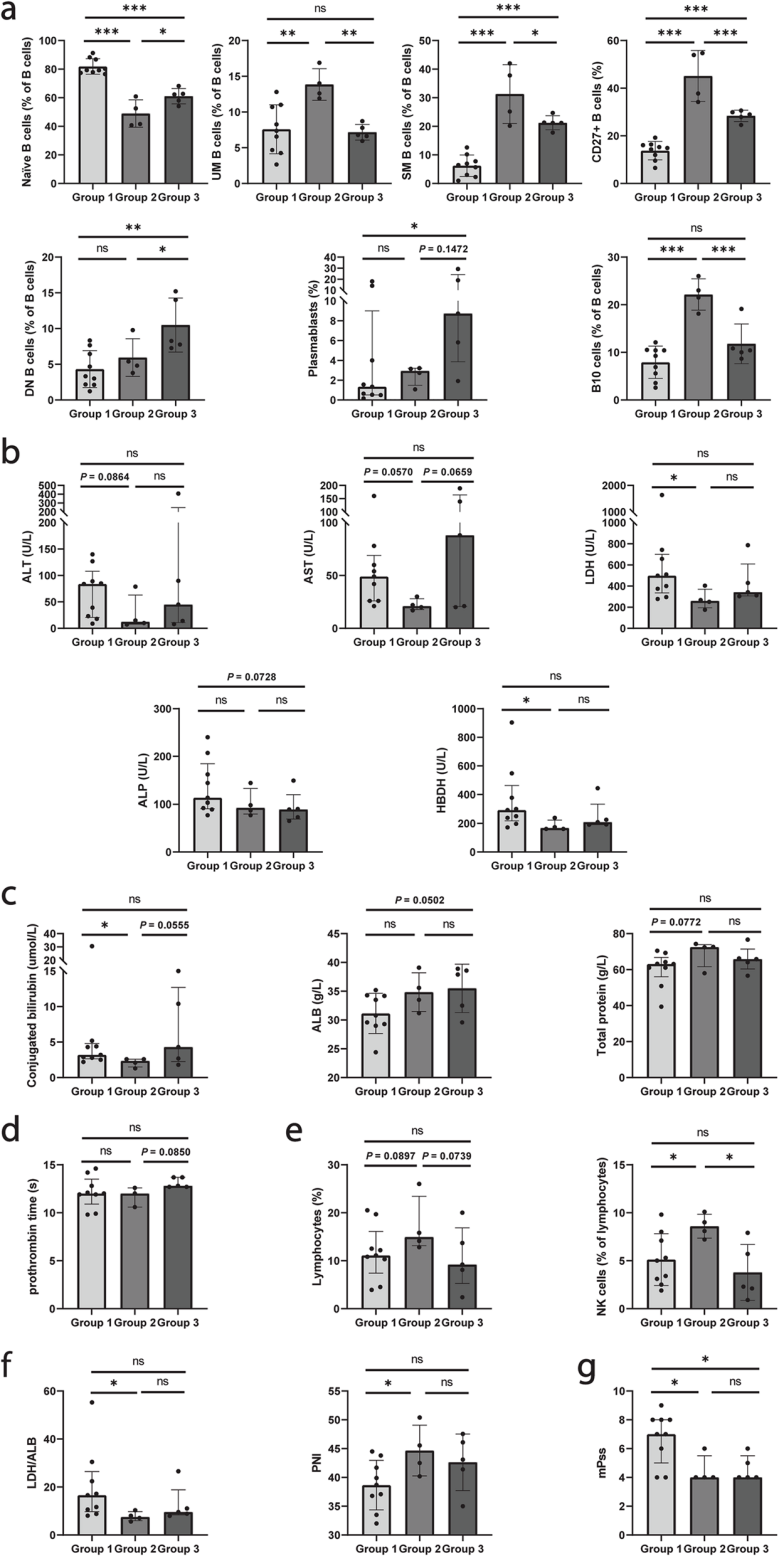
Differences of B cell subsets and clinical manifestations among the clustered groups. **a** Differences of B cell subsets among the clustered groups. Differences of clinical manifestations among the clusters, including liver and myocardial enzymes (**b**), other liver function indicators (**c**), coagulation parameters (**d**), immune cells (**e**), indexes and ratios (**f**), and mPss (**g**). *P < 0.05, **P < 0.01, ***P < 0.001, and ns, no significance (Kruskal–Wallis *H* test followed by uncorrected Dunn’s test)

Then, the frequencies of B cell subsets in the three groups were compared (Fig. 5a). Group 1 showed the highest naïve B cells; however, SM B cells and total CD27^+^ memory B cells were lowest in this group. Group 2 had the highest UM B cells, SM B cells, total CD27^+^ B cells, and B10 cells. In addition, group 3 had the highest DN B cells and plasmablasts [25], with lower UM B cells and B10 cells. SM B cells and total CD27^+^ B cells are higher in group 3 than group 1, while UMB cells and B10 cells have no significant differences between group 1 and group 3.

Finally, the clinical manifestations of the three groups were compared (Fig. 5b–g, Tables S2 and S3). Liver function-related indicators are compared among groups. Higher liver enzymes were shown in group 1, while group 2 had relatively low liver enzymes. Group 3 also had a higher AST level than group 2 (Fig. 5b). Group 2 also had the lowest conjugated bilirubin (CB), and group 1 had lower ALB and serum total protein (TP) than group 2 and group 1, respectively (Fig. 5c). In coagulation parameters, group 2 had shorter prothrombin time (PT) than group 3 (Fig. 5d). Group 2 also had the highest LYM% and NK% (Fig. 5e). In inflammatory indexes and ratios, group 1 had higher LDH/ALB (LAR) and lower PNI than group 2 (Fig. 5f). In addition, group 1 also had higher mPss than group 2 and group 3 (Fig. 5g).

## Discussion

In this study, we explored the B cell subset features in AOSD patients for the first time. B cell subsets’ frequencies between AOSD patients and HCs were significantly different. In addition, some B cell subsets were correlated to AOSD-related manifestations. These manifestations include frequencies of immune cells, liver function parameters, coagulation parameters, and inflammation-related indexes and ratios.

The unbiased cluster based on B cell subsets divided AOSD patients into group 1 (naïve B cells-dominant group), group 2 (CD27^+^ memory B cells-dominant group), and group 3 (precursors of autoantibody-producing plasma cells-dominant group). Different clinical manifestations were also found among the three groups. This result may contribute to developing B cell-based AOSD classification and personalized treatments.

Alterations of the frequencies of neutrophils, monocytes, and NK cells in AOSD are confirmed by former research. Although no data shows a frequency change of total B cells in AOSD patients, a wider distribution was found in this study and former research [12], indicating B cell abnormalities occurred at least in some AOSD cases. Alterations of the frequencies of B cell subsets indicate their potential roles in AOSD. The increased B cell subsets, including naive B cells, DN B cells, and plasmablasts, may promote the development of the AOSD. In the same way, the decreased B cell subsets, such as UM B cells and B10 cells, may play immune regulatory roles in AOSD. The functions of UM B cells and B10 cells have been partially revealed in other rheumatic diseases [24–28].

Because lymphopenia is one of the manifestations of systemic inflammation [22], the positive correlation between the two B cell subsets (UM cells and B10 cells) and LYM% may mean a lower disease activity in patients with higher UM B and B10 cells, which is consistent with the potential immune regulatory function of these cells. Since NK% is lower in acute AOSD [29], a negative correlation between naïve B cells and NK cells may indicate higher disease activity in patients with a higher level of naïve B cells. The positive correlation between naïve B cells and B% indicates the potential disease-promoting function of B cells in AOSD. However, DN B cells and plasmablasts negatively correlated with B%. This phenomenon may reflect the heterogeneity of AOSD patients.

Liver abnormalities are common (65%) in AOSD patients [1]. Higher naïve B cells and lower UM B cells, SM B cells, total CD27^+^ memory B cells, DN B cells, and B10 cells are shown in AOSD patients with abnormalities of parameters related to liver function. This result is partly consistent with a study about liver cirrhosis [30]. Since liver enzyme abnormalities also occur in other rheumatic diseases [31], the mechanisms behind B cell subset alterations in AOSD and these diseases should be further studied. In addition, the reason why DN B cells negatively correlated with liver enzymes such as ALP needs to be found.

Moreover, the correlation between B cell subsets and coagulation may also be related to liver function abnormalities. Since disseminated intravascular coagulation (DIC) may occur in AOSD [32], the relationship between B cell subsets and coagulation is also worth attention. Systemic immune-inflammation indexes and ratios also correlated with B cell subsets, while the single parameters compositing these indexes did not. The efficient applications of indexes and ratios showed an advantage of composite indicators in AOSD and other diseases assessment and research.

About 50% of patients were clustered into group 1 (naïve B cells-dominant group) and showed relatively higher liver enzymes and bilirubin, with the highest mPss. Clinical manifestations of patients in group 2 (CD27^+^ memory B cells-dominant group) are relatively normal compared to other groups, which might attribute to the immune regulatory function of UM B cells and B10 cells. Patients in group 3 (precursors of autoantibody-producing plasma cells-dominant group) had higher AST and lower ALP, with prolonged PT than group 2. Group 3 showed a distinct disease pattern related to liver and coagulative dysfunction, although the mPss is not very high. Since precursors of autoantibody-producing plasma cells are dominant in group 3, autoantibodies might play more critical roles in the pathogenesis of group 3 cases.

Our study has several limitations. Firstly, the sample size was small since only 27 patients and 18 active ones were studied. Then, only one of the patients was male, and the gender distribution may affect the results. Next, only one of the patients had not been treated with corticosteroids or immunosuppressants before sample collection. Therefore, more AOSD patients, especially medication-free ones, should be enrolled to reduce the biases and confirm the results. In addition, other B cell-related features, such as antibodies and cytokines, also need to be further studied.

## Conclusion

This study preliminarily describes the peripheral B cell subset features in AOSD patients. The B cell subset alterations help reflect the disease status of AOSD, especially in immune cells, liver function, and nutrition. On the other hand, B cell subsets may also be used to classify AOSD due to their good performance in clustering and correlation analysis. The abnormal B cell subset distribution in AOSD can also inspire targeting B cell therapy, but the mechanisms behind it need to be clarified before going further.

## Supplementary Information

**Additional file 1: Fig. S1.** B cell subset alteration in AOSD compared to HCs. (a) Results of PCA based on B cell subsets in HCs and the active & inactive AOSD patients, first and second principal components were chosen to virtualize different B cell subsets. (b) Comp1 and Comp 2 values in individual patients with AOSD.

**Additional file 2: Table S1.** Subgroup analyses of B cell subsets according to clinical features.

**Additional file 3: Table S2.** Difference in clinical manifestations among three groups.

**Additional file 4: Table S3.** Clinical characteristics of three groups.

## Data Availability

The datasets used and/or analyzed during the present study are available from the corresponding author on reasonable request.

## Abbreviations

AOSD: Adult-onset Still’s disease
HCs: Healthy controls
mPss: Modified Pouchot systemic score
UM B cells: IgD^+^CD27^+^ unswitched memory B cells
SM B cells: IgD^−^CD27^+^ switched memory B cells
DN B cells: IgD^−^CD27^−^ double negative B cells
B10 cells: CD24^hi^CD27^+^ B cells
PCA: Principal component analysis
B%: Percentage of B cells in lymphocytes
NK%: Percentage of NK cells in lymphocytes
LYM%: Percentage of lymphocytes
PT: Prothrombin time
APTT: Activated partial thromboplastin time
ALT: Alanine transaminase
AST: Aspartate transaminase
GGT: γ-Glutamyl transferase
ALP: Alkaline phosphatase
HBDH: Hydroxybutyrate dehydrogenase
LDH: Lactate dehydrogenase
ALB: Serum albumin
LAR: LDH/ALB ratio
PNI: Prognostic nutritional index
TG: Triglyceride
CB: Conjugated bilirubin
TP: Serum total protein
DIC: Disseminated intravascular coagulation
WBC: White blood cell
SII: Systemic immune-inflammation index
NLR: Neutrophil count/lymphocyte count ratio
