# Supplementary figures and images for: B cell subsets in adult-onset Still’s disease: potential candidates for disease pathogenesis and immunophenotyping
Source: Arthritis Res Ther. 2023 Jun 15;25:104. doi: 10.1186/s13075-023-03070-2 (PMC10268358; doi:10.1186/s13075-023-03070-2)

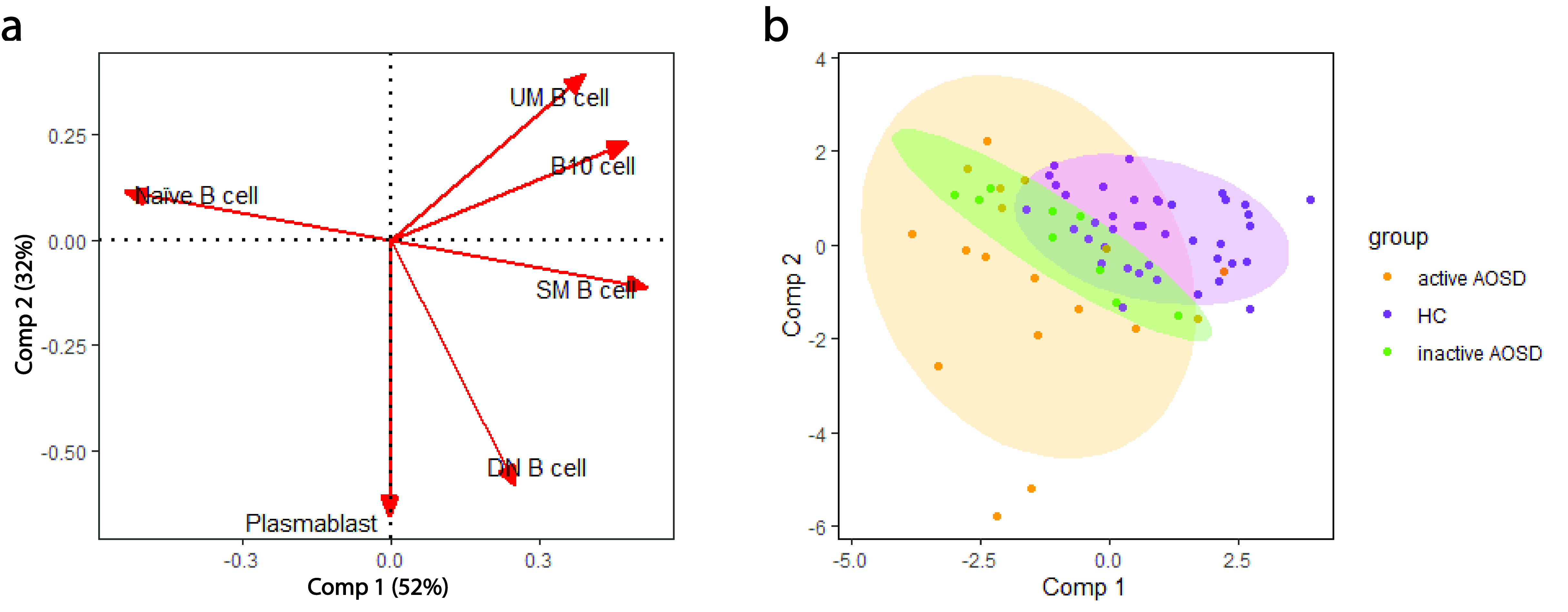

Supplement: Supplementary file 1 — Additional file 1: Fig. S1. B cell subset alteration in AOSD compared to HCs. (a) Results of PCA based on B cell subsets in HCs and the active & inactive AOSD patients, first and second principal components were chosen to virtualize different B cell subsets. (b) Comp1 and Comp 2 values in individual patients with AOSD. [file 13075_2023_3070_MOESM1_ESM.tif]
