# Supplementary material for: B cell subsets in adult-onset Still’s disease: potential candidates for disease pathogenesis and immunophenotyping
Source: Arthritis Res Ther. 2023 Jun 15;25:104. doi: 10.1186/s13075-023-03070-2 (PMC10268358; doi:10.1186/s13075-023-03070-2)
Supplement: Supplementary file 2 — Additional file 2: Table S1. Subgroup analyses of B cell subsets according to clinical features. [file 13075_2023_3070_MOESM2_ESM.docx]

**Table S1 Subgroup analyses of B cell subsets according to clinical features**

|  |  | Naïve B cells | UM B cells | SM B cells | CD27^+^ B cells | DN B cells | Plasmablasts | B10 cells |
| --- | --- | --- | --- | --- | --- | --- | --- | --- |
| Fever | Median  *P* value | 69.6 vs 73.1  1.000 | 7.2 vs 10.1  0.247 | 16.9 vs 12.8  0.965 | 22.1 vs 21.9  0.829 | 7.7 vs 5.1  0.515 | 3.7 vs 2.9  0.762 | 10.4 vs 11.3  0.762 |
| Arthritis | Median  *P* value | 77.4 vs 62.7  0.659 | 7.4 vs 8.3  0.375 | 10.2 vs 20.2  0.536 | 15.3 vs 27.9  0.375 | 4.8 vs 5.8  0.375 | 3.2 vs 2.7  0.930 | 9.3 vs 10.4  0.246 |
| Skin rash | Median  *P* value | 62.3 vs 79.2  0.328 | 8.7 vs 7.8  0.246 | 20.2 vs 7.8  0.211 | 27.9 vs 16.1  0.596 | 5.4 vs 6.4  0.328 | 3.1 vs 1.9  0.179 | 10.8 vs 10.0  0.596 |
| Sore throat | Median  *P* value | 78.9 vs 62.5  0.125 | 6.6 vs 8.5  0.125 | 7.9 vs 20.7  0.102 | **15.1 vs 29.5**  **0.041*** | 5.5 vs 5.9  0.682 | 1.5 vs 3.2  0.553 | 9.8 vs 11.5  0.151 |
| Splenomegaly | Median  *P* value | 78.4 vs 65.3  0.180 | 7.2 vs 8.5  0.213 | 9.1 vs 19.0  0.180 | 15.8 vs 27.3  0.151 | 5.8 vs 5.9  0.892 | 11.4 vs 2.3  0.250 | 9.0 vs 11.5  0.053 |
| Lymphadenopathy | Median  *P* value | 72.4 vs 69.9  0.750 | 8.0 vs 8.1  0.892 | 15.2 vs 15.2  0.964 | 20.5 vs 24.6  0.820 | 5.0 vs 7.1  0.494 | 2.5 vs 3.4  1.000 | 10.4 vs 11.5  0.892 |
| Serositis | Median  P value | 68.2 vs 76.5  0.605 | 7.8 vs 8.3  0.796 | 17.8 vs 12.6  0.436 | 24.6 vs 16.4  0.666 | 4.8 vs 7.7  0.161 | 2.7 vs 3.2  1.000 | 10.4 vs 10.4  0.931 |

The medians (presence vs absence) and P value are shown in the table. The data was analyzed by Mann–Whitney *U* test, *P < 0.05.

Abbreviations: UM B cells: IgD^+^CD27^+^ unswitched memory B cells; SM B cells: IgD^-^CD27^+^ switched memory B cells; DN B cells: IgD^-^CD27^-^ double negative B cells; B10 cells: CD24^hi^CD27^+^ B cells.
