# Supplementary material for: B cell subsets in adult-onset Still’s disease: potential candidates for disease pathogenesis and immunophenotyping
Source: Arthritis Res Ther. 2023 Jun 15;25:104. doi: 10.1186/s13075-023-03070-2 (PMC10268358; doi:10.1186/s13075-023-03070-2)
Supplement: Supplementary file 3 — Additional file 3: Table S2. Difference in clinical manifestations among three groups. [file 13075_2023_3070_MOESM3_ESM.docx]

**Table S2 Difference in clinical manifestations among three groups**

| **Clinical manifestations** | ***P* value** |
| --- | --- |
| Age  Disease duration  mPss  WBC, ×109/L  Percentage of neutrophils, %  Percentage of lymphocytes, %  Percentage of monocytes, %  Neutrophil count, x109/L  Lymphocyte count, x109/L  Monocyte count, x109/L  Percentage of T cells, %  Percentage of B cells, %  Percentage of NK cells, %  Hb, g/L  PLT, ×109/L  ALT, U/L  AST, U/L  AST/ALT  GGT, U/L  LDH, U/L  ALP, U/L  HBDH, U/L  TBIL, mmol/L  CBIL, mmol/L  TG, mmol/L  TP, g/L  ALB, g/L  GLB, g/L  AGR  Fibrinogen, g/L  D-Dimer, μg/L  PT, sec  APTT, sec  ESR, mm/H  CRP, mg/L  Ferritin, μg/L  SII  CAR  PNI  FER  NLR  LAR | 0.259  0.369  **0.038***  0.917  0.320  0.153  0.831  0.861  0.361  0.751  0.458  0.311  **0.035***  0.235  0.329  0.223  0.115  0.736  0.266  0.073  0.163  **0.049***  0.235  0.083  0.616  0.181  0.116  0.569  0.581  0.569  0.434  0.156  0.457  0.542  0.937  0.291  0.510  0.861  0.105  0.165  0.310  **0.036*** |

The data was analyzed by Kruskal-Wallis *H* test. *P < 0.05.

Abbreviations: mPss: modified Pouchot score; WBC: white blood cell; Hb: hemoglobin; PLT: platelet count; ALT: alanine aminotransferase; AST: aspartate aminotransferase; GGT: γ-glutamyl transpeptidase; LDH: lactate dehydrogenase; ALP: alkaline phosphatase; HBDH: hydroxybutyrate dehydrogenase; TBIL: total bilirubin; CBIL: conjugated bilirubin; TG: triglycerides; TP: total protein; ALB: albumin; GLB: globulin; AGR: albumin/globulin ratio; PT: prothrombin time; APTT: activated partial thromboplastin time; ESR: erythrocyte sedimentation rate; CRP: C-reactive protein; RF: rheumatoid factor; ANA: antinuclear antibody; ACPA: anti-cyclic citrullinated peptide antibody. SII: systemic immune-inflammation index (PLT × neutrophil count/lymphocyte count); CAR: CRP/ALB ratio; PNI: prognostic nutritional index (albumin + 0.005 × peripheral lymphocyte count); FER: ferritin/ESR ratio; NLR: neutrophil count/lymphocyte count ratio; LAR: LDH/ALB ratio.
