# Supplementary material for: B cell subsets in adult-onset Still’s disease: potential candidates for disease pathogenesis and immunophenotyping
Source: Arthritis Res Ther. 2023 Jun 15;25:104. doi: 10.1186/s13075-023-03070-2 (PMC10268358; doi:10.1186/s13075-023-03070-2)
Supplement: Supplementary file 4 — Additional file 4: Table S3. Clinical characteristics of three groups. [file 13075_2023_3070_MOESM4_ESM.docx]

**Table S3 Clinical characteristics of three groups**

| Characteristics | Group 1 (n = 9) | Group 2 (n = 4) | Group 3 (n = 5) |
| --- | --- | --- | --- |
| Fever (n, %)  Arthritis (n, %)  Skin rash (n, %)  Sore throat (n, %)  Splenomegaly (n, %)  Lymphadenopathy (n, %)  Liver dysfunction (n, %)  Serositis (n, %)  mPss ≥ 6 (n, %)  WBC ≥ 15 × 10^9^/L (n, %)  Ferritin ＞ 1500 μg/L (n, %) | 5 (55.6)  5 (55.6)  6 (66.7)  5 (55.6)  4 (44.4)  6 (66.7)  8 (88.9)  4 (44.4)  6 (66.7)  5 (55.6)  7 (77.8) | 1 (25.0)  1 (25.0)  3 (75.0)  0 (0.0)  0 (0.0)  3 (75.0)  2 (50.0)  2 (50.0)  1 (25.0)  2 (50.0)  2 (50.0) | 4 (80.0)  1 (20.0)  3 (60.0)  1 (20.0)  2 (40.0)  3 (60.0)  3 (60.0)  3 (60.0)  2 (40.0)  2 (40.0)  3 (60.0) |
